# Supplementary figures and images for: Sage extract and ascorbic acid derivative inhibit melanogenesis via downregulating keratinocyte-derived GM-CSF
Source: PLoS One. 2025 Jun 10;20(6):e0325242. doi: 10.1371/journal.pone.0325242 (PMC12151418; doi:10.1371/journal.pone.0325242)

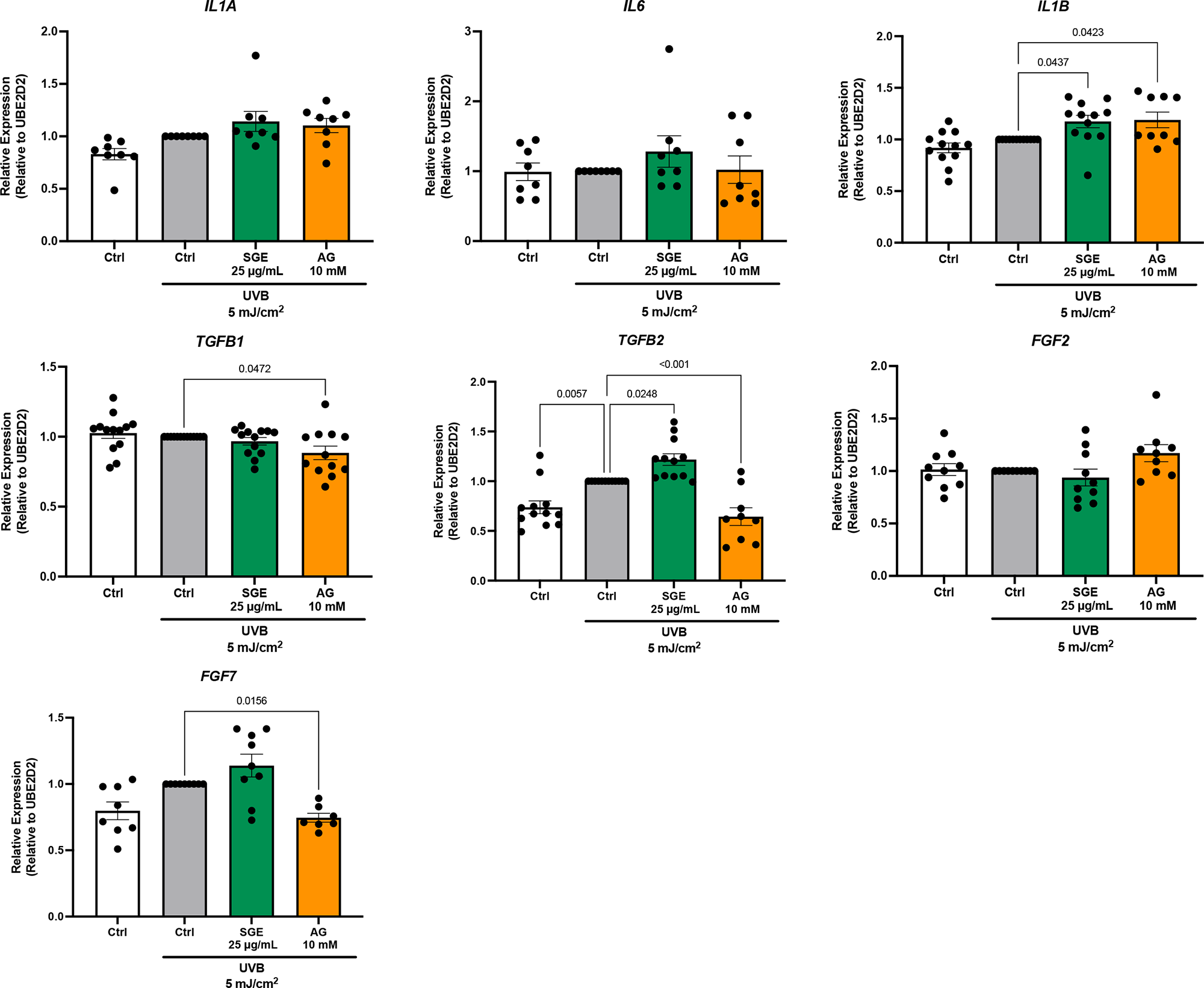

Supplement: S1 Fig — HPEKs were treated with either SGE (25 µg/mL) or AG (10 mM), incubated for 30 min, and then exposed to UVB irradiation at a dose of 5 mJ/cm2. Subsequently, co-culturing with NHEMs was initiated in CnT-PR medium. Forty-eight hours after starting the co-culture, the following experiments were performed. Gene expression levels of IL1A, IL1B, IL6, FGF2, FGF7, TGFB1, and TGFB2 were quantified by q-PCR analysis. Details of the primers used in these experiments are shown in S2 Table. The graphs indicate the mean ± SEM values for relative expression from eight independent experiments. Statistical significance was calculated using one-way ANOVA followed by Dunnett’s test. (TIF) [file pone.0325242.s001.tif]

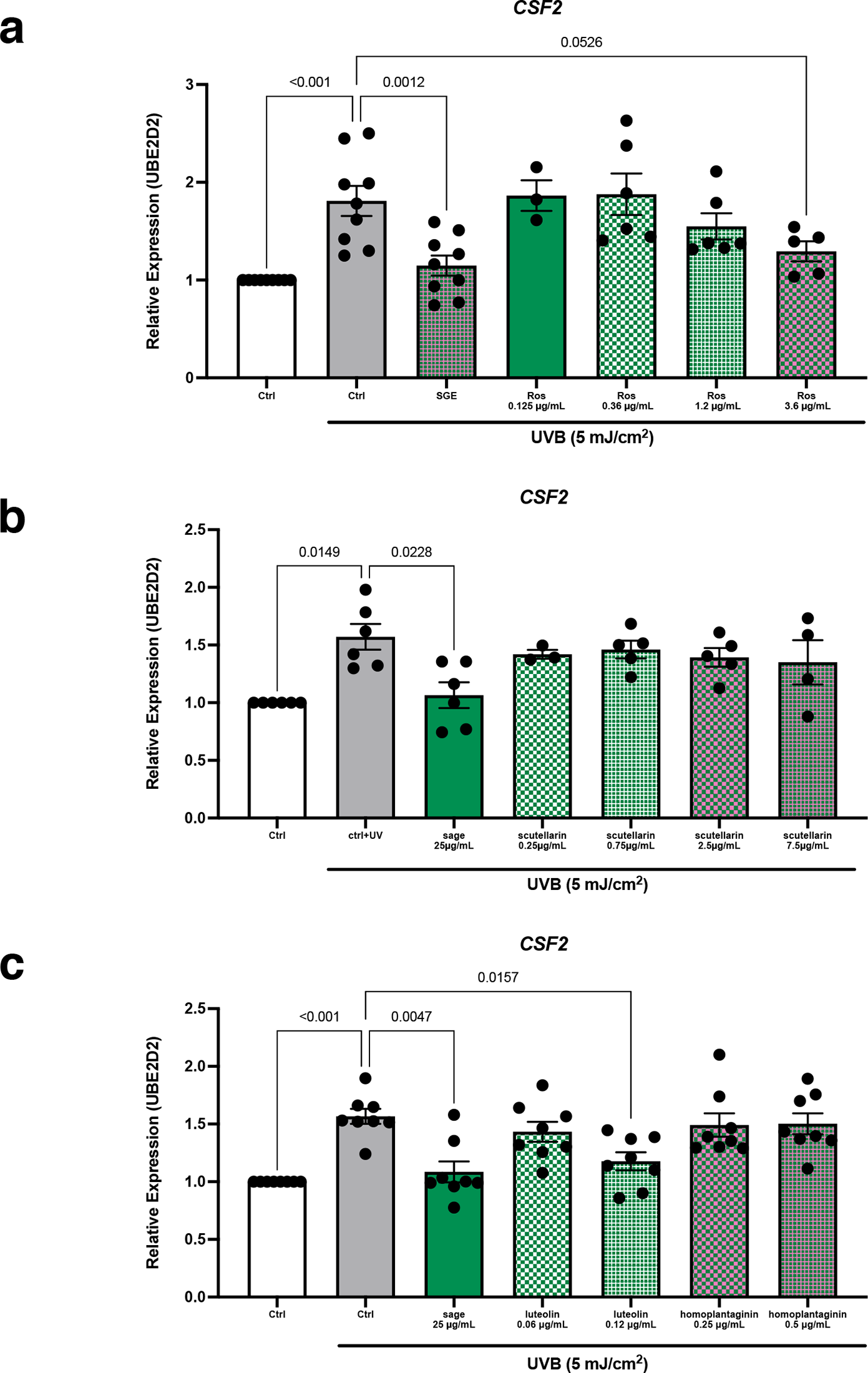

Supplement: S2 Fig — q-PCR analysis of CSF2 expression in HPEKs. HPEKs were exposed to UVB radiation (5 mJ/cm2) and incubated for 72 h. (a) HPEKs were treated with either SGE (25 µg/mL) or rosmarinic acid (0.125, 0.36, 1.2, 3.6 μg/mL) 30 min before UVB exposure. (b) HPEKs were treated with either SGE or scutellarin (0.25, 0.75, 2.5, 7.5 μg/mL) 30 min before UVB exposure. (c) HPEKs were treated with SGE, luteorin (0.06, 0.12 μg/mL), or homoplantaginin (0.25, 0.50 μg/mL) 30 min before UVB exposure. The graphs indicate the mean ± SEM values for relative expression from four to eight independent experiments. Statistical significance was calculated using one-way ANOVA followed by Dunnett’s test. (TIF) [file pone.0325242.s002.tif]

**Fig 4** Those are the final figures.

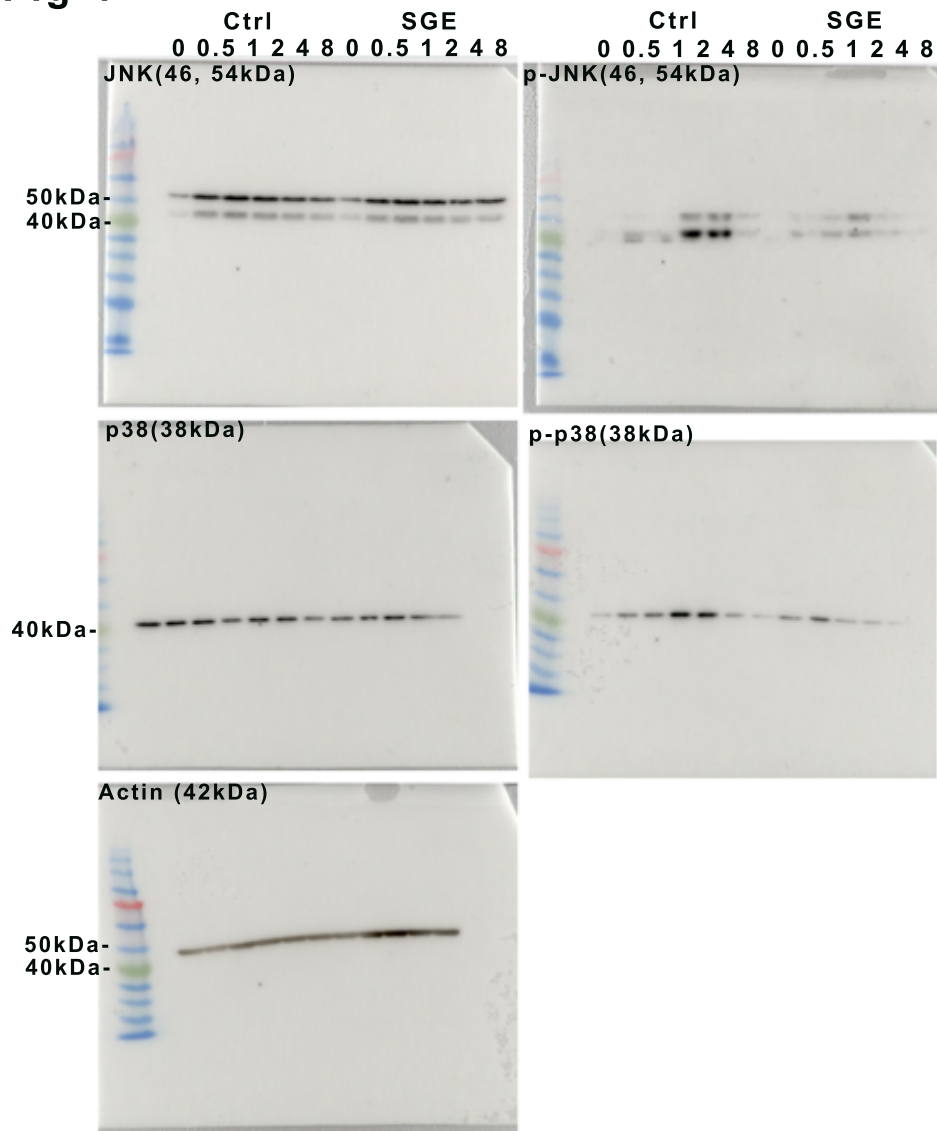

Supplement: S1_raw_images — Original images of protein Western blot experiments used in the main figures. (PDF) [file pone.0325242.s005.pdf]
