# Supplementary material for: Sage extract and ascorbic acid derivative inhibit melanogenesis via downregulating keratinocyte-derived GM-CSF
Source: PLoS One. 2025 Jun 10;20(6):e0325242. doi: 10.1371/journal.pone.0325242 (PMC12151418; doi:10.1371/journal.pone.0325242)
Supplement: S1 Table — Approximately 80 natural extracts that can be utilized in quasi-pharmaceutical cosmetic products were tested in a preliminary screening for this study. These extracts were obtained safe extraction solution (ex. water, ethanol, 1,3-butylene glycol) for skin followed by freeze drying. The 3D-skin model was cultured for 11 days. UV-B irradiation, medium replacement, and the addition of PBS containing the natural extracts (0.1 μg/mL) were performed every other day. Melanin contents were measured following 11 days culture. (PDF) [file pone.0325242.s003.pdf]

	Extract Name	Solvent	Melanin Contents / Control (%)
1	Actinidia Chinensis (Kiwi) Fruit Extract	Ethanol	N.E.
2	Aloe Ferox Leaf Extract	Ethanol	N.E.
3	Alpinia Katsumadai Seed Extract	Butylene Glycol	98.60%
4	Alpinia Leaf Extract	Butylene Glycol	N.E.
5	Angelica Extract	Butylene Glycol	N.E.
6	Betula Platyphylla Japonica Bark Extract	Ethanol	N.E.
7	Bitter Orange Extract	Ethanol	N.E.
8	Camellia Sinensis Leaf Extract	Butylene Glycol	N.E.
9	Citrus Aurantium Amara (Bitter Orange) Peel Extract	Butylene Glycol	N.E.
10	Citrus Grandis (Grapefruit) Fruit Extract	Ethanol	N.E.
11	Citrus Reticulata (Tangerine) Peel Extract	Butylene Glycol	91.06%
12	Clove Extract	Ethanol	N.E.
13	Coptis Chinensis Rhizome Extract	Butylene Glycol	N.E.
14	Crataegus Oxyacantha Fruit Extract	Ethanol	N.E.
15	Eisenia Arborea Extract	Ethanol	N.E.
16	Eriobotrya Japonica Leaf Extract	Ethanol	N.E.
17	Eugenia Caryophyllus (Clove) Flower Extract	Ethanol	N.E.
18	Foeniculum Vulgare (Fennel) Fruit Extract	Ethanol	N.E.
19	Fruits Wrinkle Protect Essence	juice	N.E.
20	Ganoderma Lucidum (Mushroom) Stem Extract	water	N.E.
21	Gardenia Florida Fruit Extract	Butylene Glycol	N.E.
22	Gentian Extract	Butylene Glycol	N.E.
23	Gentiana Lutea Rhizome/Root Extract	Butylene Glycol	93.6%
24	Geranium Thunbergii Flower/Leaf/Stem Extract	Butylene Glycol	N.E.
25	Ginger Tincture	Ethanol	N.E.
26	Ginseng Extract	Ethanol	N.E.
27	Glycine Max (Soybean) Seed Extract	Ethanol	N.E.
28	Glycyrrhiza Extracted Powder	water	N.E.
29	Glycyrrhiza Glabra (Licorice) Leaf Extract	Butylene Glycol	N.E.
30	Golden Silk Extract	pulp	N.E.
31	Horse Chestnut Extract	Butylene Glycol	N.E.
32	Houttuynia Cordata Extract	Ethanol	N.E.
33	Hydrolyzed Conchiolin Protein	Ethanol	N.E.
34	Hypericum Perforatum Flower/Leaf/Stem Extract	Butylene Glycol	N.E.
35	Iris Florentina Root Extract	Ethanol	N.E.
36	Isodon Extract	Ethanol	N.E.
37	Isodonis Japonicus Leaf/Stalk Extract	Butylene Glycol	N.E.
38	Japanese Angelica Root Extract	Butylene Glycol	N.E.
39	Japanese Honeysukle Extract	Ethanol	N.E.
40	Japanese Honeysukle Flower Extract	Ethanol	N.E.
41	Jujube Extract	Butylene Glycol	N.E.
42	Kidachi Aloe Extract	Ethanol	N.E.
43	Linden Extract	Ethanol	N.E.
44	Loquat Leaf Extract	Ethanol	N.E.
45	Luffa Extract	Ethanol	N.E.
46	Marigold Extract	Butylene Glycol	N.E.
47	Morus Alba Root Extract	Ethanol	N.E.
48	Mulberry Extract	Butylene Glycol	N.E.
49	Natto Gum	Ethanol	N.E.
50	Noni Juice	juice	N.E.

N.E. means "No effect"

	Extract Name	Solvent	Melanin Contents / Control (%)
51	Paeonia Lactiflora Root Extract	Butylene Glycol	N.E.
52	Peach Kernel Extract	Ethanol	N.E.
53	Pearl Protein Extract	Butylene Glycol	N.E.
54	Peony Root Extract	Butylene Glycol	N.E.
55	Peony Root Extract	Ethanol	N.E.
56	Peppermint Extract	Butylene Glycol	N.E.
57	Perilla Ocymoides Leaf Extract	Ethanol	N.E.
58	Pineapple Extract	Ethanol	N.E.
59	Piper Longum Fruit Extract	juice	N.E.
60	Poria Cocos Extract	Ethanol	N.E.
61	Prune Extract	pulp	N.E.
62	Prunus Yedoensis Leaf Extract	Butylene Glycol	N.E.
63	Rehmannia Chinensis Root Extract	Butylene Glycol	N.E.
64	Rice Bran Ferment Extract	pulp	N.E.
65	Rose Extract	water	N.E.
66	Rose Myrtle Extract	Ethanol	N.E.
67	Royal Jelly Extract	Ethanol	N.E.
68	Safflower Extract	Ethanol	N.E.
**69**	**Salvia Officinalis (Sage) Leaf Extract**	**Ethanol**	**90.2%**
70	Sanguisorba Officinalis Root Extract	Butylene Glycol	N.E.
71	Saxifraga Stolonifera Extract	Ethanol	N.E.
72	Shiitake Extract	Butylene Glycol	N.E.
73	Sweet Flag Extract	Ethanol	N.E.
74	Thymus Serpillum Extract	Butylene Glycol	N.E.
75	Ume Extract	pulp	N.E.
76	Zanthoxylum Piperitum Peel Extract	Ethanol	N.E.
77	Zingiber Officinale (Ginger) Rhizome Extract	Ethanol	N.E.

N.E. means "No effect"
