# Supplementary material for: Sage extract and ascorbic acid derivative inhibit melanogenesis via downregulating keratinocyte-derived GM-CSF
Source: PLoS One. 2025 Jun 10;20(6):e0325242. doi: 10.1371/journal.pone.0325242 (PMC12151418; doi:10.1371/journal.pone.0325242)
Supplement: S2 Table — (PDF) [file pone.0325242.s004.pdf]

Name	Forward primer (5’-3’)	Reverse primer (5’-3’)
IL1A	GAATGACGCCCTCAATCAAAGT	TCATCTTGGGCAGTCACATACA
IL1B	CAGCTACGAATCTCCGACCAC	GGCAGGGAACCAGCATCTTC
IL6	AAATTCGGTACATCCTCGACGG	GGAAGGTTCAGGTTGTTTTCTGC
FGF2	ATCAAAGGAGTGTGTGCTAACC	ACTGCCCAGTTCGTTTCAGTG
FGF7	TCCTGCCAACTTTGCTCTACA	CAGGGCTGGAACAGTTCACAT
TGFB1	CAAGCAGAGTACACACAGCAT	TGCTCCACTTTTAACTTGAGCC
TGFB2	CAGCACACTCGATATGGACCA	CCTCGGGCTCAGGATAGTCT
