# Supplementary material for: Sage extract and ascorbic acid derivative inhibit melanogenesis via downregulating keratinocyte-derived GM-CSF
Source: PLoS One. 2025 Jun 10;20(6):e0325242. doi: 10.1371/journal.pone.0325242 (PMC12151418; doi:10.1371/journal.pone.0325242)
Supplement: Attachment — Submitted filename: Response to editor.docx [file pone.0325242.s008.docx]

**Response to Editor**

**Response to Editor:**

We wish to express our appreciation to the editor for their insightful comments on our paper. The comments have helped us significantly improve the paper.

**1. The authors agreed that what is reported is additive rather than synergistic. However, the title of Figure 1 still maintains synergistic. I will suggest that all synergistic effects in the manuscript be changed to additive effects since there was no fold decrease in the melanin levels.**

Response: We thank the editor for this comment. In accordance with the editor’s comment, we have changed this text to:

***Fig 1. SGE and AG additively reduce melanin production.***

**2. In the materials and methods section, “Other extracts using screening were shown in S1 Table” should be revised to “Other extracts used in the screening are shown in S1 Table.”**

Response: Thank you for pointing this out. This error has been corrected in accordance with the editor's comment.

Thank you again for your comments on our paper. We trust that the revised manuscript is suitable for publication.
